# Socio-economic differences in life expectancy among persons with diabetes mellitus or myocardial infarction: results from the German MONICA/KORA study

**DOI:** 10.1186/1471-2458-10-135

**Published:** 2010-03-16

**Authors:** Laura Perna, Uta Thien-Seitz, Karl-Heinz Ladwig, Christa Meisinger, Andreas Mielck

**Affiliations:** 1 Institute of Health Economics and Health Care Management, Helmholtz Zentrum München, German Research Centre for Environmental Health, Neuherberg, Germany; 2 City of Munich, Department of Statistics, Munich, Germany; 3 Institute of Epidemiology, Helmholtz Zentrum München, German Research Centre for Environmental Health, Neuherberg, Germany

## Abstract

**Background:**

Differences in life expectancy (LE) between social groups in a specific country are a fundamental measure of health inequalities within that country. Constant monitoring of these differences provides important information on the population's general health. The purpose of the present study is to explore and quantify the socio-economic differences in LE in Germany, focussing on a topic rarely assessed in other studies, the dependency of these LE differences on the presence of myocardial infarction or diabetes mellitus.

**Methods:**

The dataset consists of 13,427 participants (6,725 men, 6,702 women) aged 25-74 years, recruited in the region of Augsburg in Germany through three independent cross-sectional representative surveys conducted in 1984/85, 1989/90, 1994/95, with a mortality follow up in 1998 and 2002. We use a parametric model for the survival function based on the Weibull distribution, in which the hazard function is described in terms of two parameters. We estimate these parameters with a maximum likelihood method that takes into account censoring and data truncation.

**Results:**

The difference in LE between the lowest and the highest socio-economic group is estimated to be 3.79 years for men and 4.10 years for women. Diabetes mellitus reduces LE of men from the upper three income quartiles by 4.88 years, and LE of men belonging to the lowest income quartile by 7.97 years. For women, the corresponding figures are 5.79 and 5.72 years. Myocardial infarction reduces LE of men and women from the upper three income quartiles by 3.65 and 3.75 years, respectively, and LE of men and women belonging to the lowest income quartile by 5.11 and 10.95 years, respectively.

**Conclusions:**

This study shows that in Germany the differences in LE by socio-economic status are comparable to those found in other European countries, and that these differences seem to increase when diabetes mellitus or myocardial infarction is present. The statistical method used allows estimates of LE with relatively small datasets.

## Background

Life expectancy (LE) is an estimate of the average number of years that a person can expect to live. It can be defined both at birth and at any later age. It reflects the mortality rates of a population as a function of age for the year for which it is calculated. As such, it is only dependent on the observed average age-specific death rates and it should not be viewed as a reflection of future mortality rates [1].

From a public health perspective, LE at birth represents a fundamental measure of a population's state of general health. Differences in LE between different social groups are a measure of health inequalities within a country. Constant observation of LE over time allows one to assess whether a health gap between different socio-economic groups exists, whether the gap widens or narrows, and whether public health initiatives are effective.

Several studies have documented differences in LE according to socio-economic status in European [2,3] and non-European countries [4-6]. The magnitude of these differences varies among the different countries. However, socio-economic differences in LE of persons with a specific disease have not been explored in any detail. Research has mostly focused on the socio-economic differences in *mortality *in patients with diseases, such as diabetes [7,8], rather than on the socio-economic differences in *life expectancy *in these populations.

While the differences in LE among different social groups are a measure of *health inequalities within a country*, the LE gap within disease groups represents a possible measure of *health inequalities within a health care system*. If socio-economic differences in LE increase when a disease such as diabetes or myocardial infarction is present, this could indicate that the health care system is not capable of reducing health inequalities among patients. While the length of the LE per se is important for estimates within a country, *relative reduction *of the LE between socioeconomic groups is important when examining disease groups.

The estimation of LE within disease groups represents a very concrete measure that health authorities can use to monitor relevant health care systems. Reports of socio-economic differences in terms of LE instead of mortality are more suitable for illustrative purposes [9], since its repercussions for the population are easier to grasp and to report. While LE is easier to understand and communicate than mortality, its estimation presents two main difficulties related to obtaining the necessary amount of data [10], and to methodical issues [11]. This might explain why the research has mostly focused on differences in mortality within disease groups and not on LE.

Estimating LE by socio-economic status would ideally require a comprehensive census of a population linking socio-economic data with the deaths and births recorded in the year of the census. In countries not allowing this kind of data-linkage system among datasets in public registers, one can only calculate LE by socio-economic status within disease groups by using other data. Scientific datasets are required which contain data on the diseases of interest and data on socio-economic status and mortality.

These data are very difficult to obtain since they require a very long mortality follow up and also have methodological problems related to the relatively small number of people in the dataset and possible measurement bias due to left-censored data. The latter arises because study participants have already reached a certain age, while mortality data for persons who deceased at younger ages is left-truncated.

In this paper we measure LE differences among persons with diabetes and myocardial infarction with data collected in Germany. This allows us to make an important contribution to the state of the research on the LE in general and in Germany in particular, where the literature on the topic is scarce and a chronological trend is lacking. While, the health gap measured by calculating the differences in LE has recently gained some attention in Germany [e.g. [12-14]], the socio-economic differences in LE of patients with myocardial infarction or diabetes mellitus have not been measured yet.

Furthermore, monitoring the impact of socio-economic differences on health is important in Germany, since the social inequality regarding the traditional socio-economic indicators, like income, educational level and professional status, is increasing in this country. Economic indicators show that income inequality has increased in recent years [15]. From a public health perspective, it is important to assess whether widening socio-economic differences are reflected in widening socio-economic gaps in life expectancy, such as seen in the UK and the USA [16,4], and in widening socio-economic gaps in life expectancy among persons with the same disease.

In Germany, there are very few datasets covering a long follow up period and including all the data necessary for a LE analysis by socioeconomic status and disease. Probably the best is provided by the dataset of the WHO MONICA study (multinational MONItoring trends and determinants in CArdiovascular disease), and KORA study (Cooperative Health Research in the Region Augsburg; following after MONICA in the same region, i.e. Augsburg). We use the MONICA/KORA dataset to explore and quantify first the socio-economic differences in LE and then the impact of diabetes and myocardial infarction on LE of people belonging to different socio-economic groups. By detailed description of the statistical method used for calculating LE, we attempt to improve the accessibility of this kind of analysis for other studies.

## Methods

### Description of the data

The MONICA project started in the early 1980s under the initiative of the World Health Organization. The aim of the project was to investigate the causes and trends of cardiovascular disease. The Augsburg region of Bavaria in Germany, whose population structure reflects that of Germany as a whole [17], served as study region of the MONICA project. In 1996, KORA was established in the Augsburg region with the goal of continuing the MONICA project and of exploring other health issues such as diabetes, allergies, health economics, genetic, and environmental questions.

The dataset used for the present analysis consists of three independent cross-sectional population-based representative surveys carried out in 1984/85 (response rate: men 80.0%, women 78.7%), 1989/90 (response rate: men 76.4%, women 77.2%), and 1994/95 (response rate: men 74.0%, women 76.8%) in the region of Augsburg within the international WHO MONICA project. The age of the participants was 25-64 years for the first survey, and 25-74 years for the last two. 13,427 persons (6,725 men, 6,702 women) took part in the surveys. In 1998 and in 2002, as part of the KORA project, the vital status of the participants was assessed through the population registries [18]. By 31st December 2002, 1,554 persons (1,032 men, 522 women) had died.

### Per capita income

In our dataset the variable 'net household income' was measured in 8 categories (all figures in DM): < 1000, 1000 to < 1500, 1500 to < 2500, 2500 to < 3500, 3500 to < 4500, 4500 to < 5500, 5500 to < 6000, > 6000. In order to calculate the income level, we first calculated the 'mid-points' of each income class. For the lowest (< 1000) and highest (> 6000) income class we calculated two-thirds and four-thirds of the corresponding limits [19], respectively. The resulting values were then divided by the number of household members, yielding the new variable 'per capita income', which was roughly divided into terciles (low, medium or high per capita income).

### Education

The dataset also includes a variable distinguishing three educational levels:

Low 'Hauptschule/Volksschule' (lower secondary school certificate)

Medium 'Realschule/Mittlere Reife' (upper secondary school certificate)

High 'Abitur/Fachabitur/Fachhochschule' (qualification for university entrance/completion of undergraduate studies)

### Socio-economic status

An important part of the literature [20] holds that the most common indicators of social status - income, education, and working position - cannot be used interchangeably as they represent different dimensions and different causal processes on the development of health outcomes. Following this approach, we separately investigated the independent contribution of income and education to the estimation of LE in Germany. As, however, we also wanted to build two very distinct socio-economic groups, such as a group ranking low (resp. high) in both education and income, we constructed an additional variable - 'socio-economic status' - defined by combining the indicators income and education level as follows:

Low low income level - plus - low educational level

Medium medium income level - plus - medium educational level

High high income level - plus - high educational level

Within each of these three socio-economic groups, the estimated LE was calculated in the total sample, separately for men and women.

The analysis of the impact of diabetes and myocardial infarction on the LE was made by comparing only the following two income groups: 'low' (< lowest quartile) on one hand and 'medium or high' (other quartiles) on the other hand, because the sub-sample of participants with diabetes mellitus and myocardial infarction (n = 542 and n = 262, respectively) was too small for a finer grading of the income groups. The small number of deceased persons in the highest education groups also prevented us from performing an additional analysis with the indicator education among people with diabetes or myocardial infarction. In the highest educational level, there were, in fact, only 11 deceased men and 3 deceased women among those with diabetes, and 9 deceased men and no deceased woman among those with a myocardial infarction.

An increasingly important aspect of social epidemiology is the impact of inhomogeneity of the socio-economic indicators in a person on the development of health outcomes [21]. We analysed the impact of this status inconsistency on the LE of two groups characterized by high income (third tercile) and low educational level (lower secondary school), and by low income (first tercile) and high educational level (qualification for university entrance/completion of undergraduate studies), respectively. However, due to the limited number of deceased cases (n = 3) among those with diabetes or myocardial infarction in the latter group, we were not able to estimate the effects of status inconsistency on the reduction of LE within this group. This is why we conducted this further analysis only among the group characterized by high income and low educational level.

### Diabetes mellitus and myocardial infarction

The data relating to diabetes mellitus and myocardial infarction were collected by asking the participants whether they had been diagnosed with diabetes (type 1 or type 2) by a physician, or whether they took medication against it (if yes, they were asked to show the medication taken), or whether they have been treated in hospital because of a myocardial infarction. A positive answer to these questions was assumed to indicate that they had diabetes or myocardial infarction. Focusing on these two diseases gave us the possibility of analysing the effect of a chronic and non-chronic disease, respectively, on the LE. As, however, diabetes is a risk factor for cardiovascular disease, this created some overlap in our analysis. In particular, 43 persons with diabetes also had a myocardial infarction.

### Statistical analysis

In this analysis, due to the limited sample size, a parametric model, the Weibull distribution, is used for the underlying distribution of survival times. In this model the hazard function increases with age and is described in terms of two parameters.

We also used a non-parametric method to check whether the basic functional shape assumed for the distribution function was reasonable or not. In particular, a non-parametric estimate of the hazard function was calculated for the present data set and the result was compared with the hazard function that is assumed in the parametric Weibull model. The test showed that the non-parametric estimate of the hazard function increased with time and is well fit by the Weibull model with a hazard increasing with time. This provides good evidence that the parametric model used in the remainder of our analysis is adequate.

For the Weibull distribution, the survival function can be written as

This function gives the probability of a person to reach an age larger than *t*. SAS^® ^provides a built-in statistical procedure (LIFEREG) for a maximum likelihood estimate of the parameters α and γ for right-censored data, which is the relevant case for this analysis since the people of the data set who are still alive effectively constitute right censored data points. More specifically, SAS^® ^actually outputs two related parameters, an "intercept" μ and a "scale" σ, which are related to α and γ by

In terms of these parameters, the survival function is given by

and the LE can be computed as

Here Γ denotes the gamma-function.

In addition, LIFEREG supports the estimation of the influence of individual variables on the life expectancy, where their influence is combined in a linear model. This assumes that the influence of the individual variables is mutually independent. For example, if we consider socio-economic status and diabetes as variables, this procedure can estimate the separate and combined impact of these two variables on life expectancy, but it cannot be used to study if the impact of diabetes varies between different socio-economic groups, as by construction such correlations are not considered in the linear model. A possible solution lies in forming separate groups for each combination of the considered variables, and in estimating LE individually for these samples. We apply this method in estimating LE for people with diabetes (unadjusted for myocardial infarction) and in estimating LE for people with myocardial infarction (unadjusted for diabetes). We also carry out a separate analysis where we estimate the LE of people with diabetes by adjusting for myocardial infarction, and vice versa. This is done by considering diabetes and myocardial infarction as additional variables of influence in maximum likelihood estimates with LIFEREG. We always consider 'sex' as one variable of influence in order to avoid that the strong difference between men and women distorts our estimates.

A limitation of our dataset is that the participants entered the study after already having reached a certain age *t*_0_. Data for people who died before *t*_0 _does not exist in the study, which constitutes a case of left data truncation. If this is simply ignored, the absolute values of the estimated life expectancies will be biased high, because the drop of the survival function from birth to age *t*_0 _has been ignored. A better method is to properly include the information about the age of the people when they entered the MONICA/KORA study. Let this age be *t*_0_. Then the probability to survive at least until age *t *> *t*_0 _is given by S*(*t*, *t*_0_) = *S*(*t*)/*S*(*t*_0_), and the probability density to die at age *t *is f*(*t*, *t*_0_) = -dS*/d*t*. When the vital status is checked a few years later, some people will have died at an age *t*_1 _in between. The others have a present age *t*_1_, and constitute right-censored data, since for them the event of death lies at some unknown time *t *> *t*_1_.

The proper log-likelihood function to observe the data set given the survival function model is hence

Maximizing this likelihood yields the correct life expectancies that are not biased due to the finite age of people entering the MONICA/KORA study. The procedure LIFEREG maximizes the same likelihood, except that the last sum in the above equation is missing. We have implemented in SAS^® ^a program that maximizes the correct likelihood, finding that it gives values for the LE about 1 year lower than what is obtained with LIFEREG. The size of this bias is hence quite moderate, which can be easily understood due to the low mortality of people at age 0-25, which is the age range entirely missing in the MONICA/KORA dataset. As we are only interested in *relative differences *in LE between the different groups studied, we for simplicity ignore the bias for the statistical analysis. Note however that this means that *absolute life expectancies *are actually about 1 year lower than all the values quoted in the following.

The statistical analysis was conducted with the help of SAS^® ^Version 9.1 (SAS Institute Inc., Cary, NC, USA)

## Results

The basic distribution of the variables is shown in table 1. It can be pointed out, for example, that low educational level is very common (about 66%), that information on income is missing for 1,799 out of 13,427 (13%), and that 4,679 participants (35%) are included in our definition of socio-economic status (i.e. combining educational level and income in three very distinct groups). Overall, 1,554 participants (12%) have died during the follow up period. Concerning participants with diabetes mellitus, this percentage is much higher (40%; i.e. 219 from 542 participants) and it is still higher for participants with myocardial infarction (46%, i.e. 121 from 262 participants).

**Table 1:** Basic distribution of the variables

Variable	Total sample		Deceased persons	
	**Mean**	**Variance**	**Mean**	**Variance**
**Age**				
- men	48.43	13.63	67.92	10.18
- women	47.87	13.48	69.93	9.51
	**N**	**%**	**N**	**%**
**Sex**				
- men	6725	50.09	1032	66.41
- women	6702	49.91	522	33.59
- total	13427			
**Per capita income (quartiles)^a^**				
- low	2841	24.43	339	26.84
- medium/high	8787	75.57	924	73.16
- missings	1799		291	
**Socioeconomic status^b^**				
- low	2946	62.96	360	76.11
- medium	762	16.29	54	11.42
- high	971	20.75	59	12.47
**Health status**				
- diabetes mellitus^c^	542	4.04	219	14.09
- missings	3		0	
- myocardial infarction^d^	262	1.95	121	7.79
- missings	2		0	

a) Low: < lowest quartile (i.e. < 667DM)

Medium/High: ≥ lowest quartile (i.e. ≥ 667DM)

b) Low: combination of low educational level and low per capita income

Medium: combination of medium educational level and medium per capita income

High: combination of high educational level and high per capita income

c) Self-report of physician diagnosis or utilization of drugs against diabetes mellitus

d) Self-report of hospitalization because of myocardial infarction

**Figure 1:**
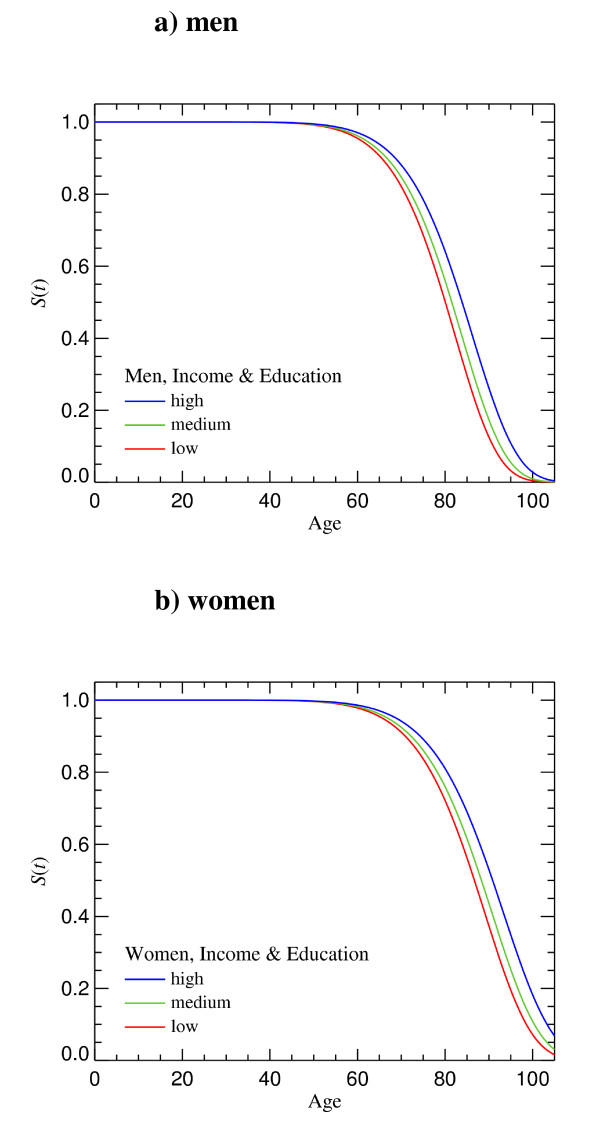
**Survival function according to socioeconomic status**. Low: combination of low educational level and low per capita income. Medium: combination of medium educational level and medium per capita income. High: combination of high educational level and high per capita income.

The results of the statistical analysis of LE show that the difference in LE between the participants in the study who have a low socio-economic status (i.e. low income - plus - low educational level) and those who have a high socio-economic status (i.e. high income - plus - high educational level) is 3.79 (82.77 vs. 78.98) years for men and 4.10 (89.63 vs. 85.53) years for women (table 2). A very similar, but more detailed association is seen in the survival curves (figures 1).

**Table 2:** Mean life expectancy at birth by socioeconomic status

	Socioeconomic status^a^	Life expectancy (years)	95% Confidence limits
**Men**	Low	78.98	76.07 - 81.99
	Medium	80.44	77.22- 83.80
	High	82.77	80.70 - 84.88
			
**Women**	Low	85.53	82.38 - 88.80
	Medium	87.11	83.62 - 90.75
	High	89.63	87.39 - 91.92

a) Low: combination of low educational level and low per capita income

Medium: combination of medium educational level and medium per capita income

High: combination of high educational level and high per capita income

The impact of diabetes on LE differs by income (table 3). On one hand, men with diabetes and higher income (upper three quartiles) have a shorter LE of 4.88 years compared to men belonging to the same income group but without diabetes (76.24 *vs. *81.12); on the other hand, men with diabetes and a lower income (lowest quartile) have a shorter LE of 7.97 years as compared to men from the same income group without diabetes (72.76 *vs. *80.73). Thus, it can be concluded that diabetes shortens the LE of poorer men considerably more than the LE of richer men, in fact, the difference amounts to about 3 more years (7.97 vs. 4.88 years). Concerning women, the reduction of LE in the diabetes group (as compared to the non diabetes group) is about 5.72 years for lower income (87.08 vs. 81.36) and 5.79 years for the higher income (87.77 vs. 81.98).

**Table 3:** Mean life expectancy at birth by income and diabetes mellitus

	Income	Diabetes mellitus	Life expectancy (years)	95% Confidence limits
**Men**	lower (lowest quartile)	Yes	72.76	68.29 - 77.52
	higher (other quartiles)	Yes	76.24	73.47 - 79.13
	lower (lowest quartile)	No	80.73	78.36 - 83.17
	higher (other quartiles)	No	81.12	79.80 - 82.46
				
**Women**	lower (lowest quartile)	Yes	81.36	77.01 - 85.96
	higher (other quartiles)	Yes	81.98	79.37 - 84.68
	lower (lowest quartile)	No	87.08	84.52 - 89.74
	higher (other quartiles)	No	87.77	86.32 - 89.25

Similar results are obtained in calculating the impact of myocardial infarction on LE (table 4). The difference in LE in the higher income group amounts to about 3.65 years for the men (80.97 years for those without a myocardial infarction *vs. *77.32 for those who had a myocardial infarction), and 3.75 years for the women (87.29 years for those without a myocardial infarction *vs. *83.54 for those with a myocardial infarction). Among the lower income group the difference is 5.11 years for the men (80.26 years for those without a myocardial infarction *vs*. 75.15 for those who had a myocardial infarction) and 10.95 years for the women (86.79 years for those without a myocardial infarction *vs*. 75.84 for those who had a myocardial infarction). Thus, myocardial infarction shortens the LE of poorer men more than the LE of richer men (5.11 *vs. *3.65 years), and a very similar but more pronounced association can also be seen for women (10.95 *vs. *3.75 years). This latter result, however, carries a large statistical uncertainty and may not be significant due to the relatively small number of women who suffered from a myocardial infarction (53 women *vs. *209 men) and died (17 women *vs. *104 men). Similarly in the case of diabetes, where the number of deceased participants among the women is almost half compared with men (79 *vs. *140).

**Table 4:** Mean life expectancy at birth by income and myocardial infarction

	Income	Myocardial infarction	Life expectancy (years)	95% Confidence limits
**Men**	lower (lowest quartile)	Yes	75.15	68.26 - 82.73
	higher (other quartiles)	Yes	77.32	72.76 - 82.18
	lower (lowest quartile)	No	80.26	78.01 - 82.58
	higher (other quartiles)	No	80.97	79.71 - 82.24
				
**Women**	lower (lowest quartile)	Yes	75.84	69.45 - 82.81
	higher (other quartiles)	Yes	83.54	78.75 - 88.63
	lower (lowest quartile)	No	86.79	84.37 - 89.27
	higher (other quartiles)	No	87.29	85.93 - 88.67

**Figure 2:**
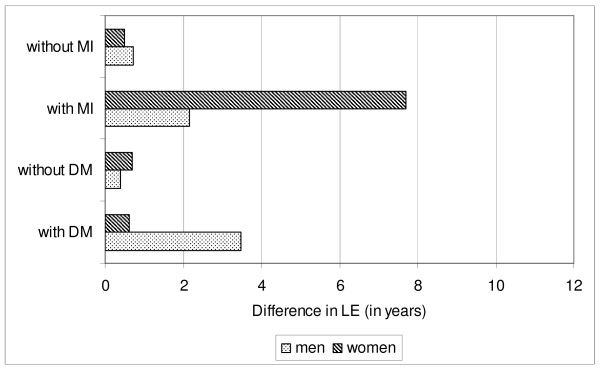
**LE of people with higher income - minus - LE of people with lower income**. LE: life expectancy; DM: diabetes mellitus; MI: myocardial infarction. lower income: lowest quartile; higher income: upper three quartiles.

**Figure 3:**
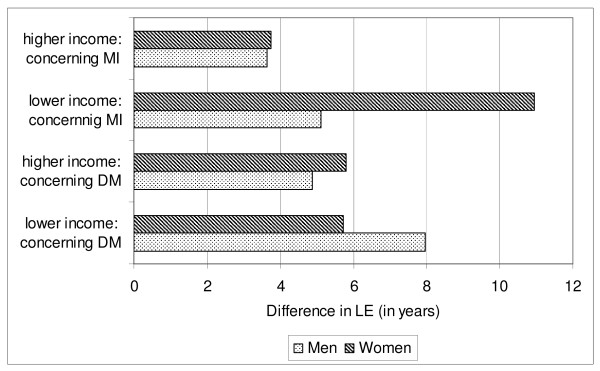
**LE of people without MI/DM - minus - LE of people with MI/DM**. LE: life expectancy; DM: diabetes mellitus; MI: myocardial infarction. lower income: lowest quartile; higher income: upper three quartiles.

The differences in LE are illustrated in figures 2 and 3. Figure 2 shows the differences in LE between the higher and the lower income group. It can be seen, for example, that this difference is greater for men and women with myocardial infarction than for men and women without myocardial infarction, and greater for men with diabetes than for men without diabetes. Figure 3 shows the differences in LE between people without resp. with myocardial infarction or diabetes. It can be seen, for example, that this difference is greater for the lower than for the higher income group, concerning men and women with (resp. without) myocardial infarction, and also concerning men with (resp. without) diabetes.

The socio-economic gap in the estimated LE remains stable both for men and women with diabetes or myocardial infarction if the impact of the other disease (i.e. myocardial infarction for people with diabetes, and vice versa) is controlled for in the logistic regression model (tables 5 and 6).

**Table 5:** Mean life expectancy at birth by income and diabetes mellitus adjusted for myocardial infarction

	Income	Diabetes mellitus	Life expectancy (years)	95% Confidence limits
**Men**	lower (lowest quartile)	Yes	73.48	68.93 - 78.33
	higher (other quartiles)	Yes	76.53	73.72 - 79.44
	lower (lowest quartile)	No	81.13	78.74 - 83.60
	higher (other quartiles)	No	81.40	80.06 - 82.75
				
**Women**	lower (lowest quartile)	Yes	81.71	77.26 - 86.42
	higher (other quartiles)	Yes	82.09	79.46 - 84.81
	lower (lowest quartile)	No	87.23	84.63 - 89.89
	higher (other quartiles)	No	87.89	86.42 - 89.38

**Table 6:** Mean life expectancy at birth by income and myocardial infarction adjusted for diabetes mellitus

	Income	Myocardial infarction	Life expectancy (years)	95% Confidence limits
**Men**	lower (lowest quartile)	Yes	76.19	69.19 - 83.90
	higher (other quartiles)	Yes	77.83	73.26 - 82.70
	lower (lowest quartile)	No	80.84	78.57 - 83.18
	higher (other quartiles)	No	81.51	80.24 - 82.81
				
**Women**	lower (lowest quartile)	Yes	77.90	70.85 - 85.64
	higher (other quartiles)	Yes	84.36	79.41 - 89.61
	lower (lowest quartile)	No	87.58	85.05 - 90.18
	higher (other quartiles)	No	87.97	86.53 - 89.41

Further analyses (not presented here in tables or figures) have been conducted looking at the educational and income levels separately. The results show that while the LE of men with a high education is approximately one year shorter than the LE of those who have high education and high income (81.96 *vs. *82.77), the LE of those belonging to the low education group is approximately one year longer than the LE of those men who have low income and low educational level (79.71 *vs. *78.98). For the women this reduction of approximately one year only applies to those belonging to the high educational level (88.37 *vs. *89.63). For the low education group there is no such difference (85.57 *vs. *85.53).

Looking just at the income, the LE of those men and women belonging to the lower income group remains almost unchanged if compared to the LE of those who have low income and low education (79.06 *vs. *78.98 and 85.95 *vs. *85.53, respectively). The LE of men and women belonging to the higher income group is instead reduced by approximately one year if compared to the LE estimate computed looking at both high income and high education (81.83 *vs. *82.77 and 88.57 *vs. *89.63, respectively).

Our results also show that the effects of status inconsistency on the LE are noticeable. Those men and women, in fact, who have an income corresponding to the lowest tercile and an education corresponding to a qualification for university entrance/completion of undergraduate studies have a LE shorter than those men and women characterized by low income plus low education. The corresponding figures are a reduction of about one and half year for the men (77.16 *vs. *78.98) and 3 years for the women (82.97 *vs. *85.53). On the contrary, those men and women characterized by an income corresponding to the highest tercile and an education corresponding to a lower secondary school have a longer LE than those having low income plus low education, and a shorter LE than those having high education and high income. The corresponding figures are 80.78 *vs. *78.98 and 80.78 *vs. *82.77 for the men, and 86.86 *vs. *85.53 and 86.86 *vs. *89.63 for the women.

The reduction of the LE among those who have diabetes or myocardial infarction and high income plus low education is not very pronounced if compared to the group of men earning more than the lowest quartile. The corresponding figures concerning the groups with diabetes are a reduction of 5 years for the men (75.67 *vs. *80.53), and 4 years for the women (82.73 *vs. *86.91). Almost unchanged are also the results for the men having myocardial infarction if compared to the group of men with high income (77.12 *vs. *80.31 and 77.32 *vs. *80.97, respectively). In the group of women, the reduction is about 2 years (84.47 *vs. *86.52).

## Discussion

The results of our study agree with the findings of a EU report in 2006 [3], including data from 21 different European countries, in which differences in LE at birth between the lowest and highest socio-economic groups are estimated to be between 4-6 years among men and 2-4 years among women. They also agree with German studies conducted with other datasets and confirm that in Germany there is a socio-economic gap in life expectancy. For example, a recent German study [14] conducted with administrative records from the German Public Health Pension System found that LE rises almost linearly with lifetime earnings - a proxy for socio-economic status. An analysis conducted with data of the LE-Survey [22] showed that while 45-year-old men, who work as "Beamte" (public officers), have a remaining LE of about 32 years, manual workers have a remaining LE of about 26 years. Among women, the LE gap between 45-year-old "Beamte" and simple employees is estimated to be about 5 years. The data of the socio-economic panel (SOEP) have provided the basis for different analyses which all showed the existence of a socio-economic gap in LE. Lauterbach et al. [23] calculated that the probability to reach retirement age for the men belonging to a lower income group (< 1,500 Euro) compared to those belonging to a higher income group (> 4,500 Euro) is 79,1% and 90,0%, respectively. Lampert et al. [13] estimated that the difference in "healthy" LE among men and women earning less than 60% of the median of the income in Germany and those earning more than 150% of the median is about 14 healthy years for the men and 10 for the women.

Possible reasons that would explain the differences in LE by socio-economic status have been amply discussed in the literature [24]. They include explanations related to different life styles among lower and higher socio-economic groups. This has also been investigated by another study using the German MONICA/KORA data [25], which showed that men and women with a higher educational level have a lower consumption of tobacco and alcohol, are more likely to engage in leisure-time physical activity, have a lower Body Mass Index, and have less job strain.

Our results also show that if German men and women with lower income have diabetes or myocardial infarction then their LE is reduced more by the disease than the LE of richer men. Adjustment for diabetes and myocardial infarction for people with myocardial infarction and diabetes, respectively, yields only very limited changes of the LE estimates. This means that the influence of the interaction of these diseases on the reduction of LE is negligible, and that the reduction in LE lies mostly in the social differences in our regression model. This indirectly confirms the results of a German study, also conducted in the region of Augsburg, which showed that the number of deceased persons with a first time myocardial infarction was about 60% higher among those belonging to a lower socio-economic group compared to those belonging to a higher socio-economic group [26]. Another German study demonstrated that persons with diabetes mellitus belonging to a lower socio-economic group are more susceptible to diabetes-specific complications, such as micro and macrovascular complications, compared to those who belong to a higher socio-economic group [27]. Clearly, a higher frequency of diabetes-specific complications could contribute to a shorter life expectancy.

Income and education seem to have similar effects on the LE of both men and women. Computation of LE based on separate analyses of income and education yielded similar estimations (79.06 *vs. *79.71 and 81.83 *vs. *81.96 for the men in the lower and higher groups, respectively, and 85.57 *vs. *85.95 and 88.57 *vs. *88.37 for the women). These figures also show that the social group with both the highest income and education is the group with the best LE in our analysis.

The group of those men and women who have an income corresponding to the lowest tercile and an education corresponding to a qualification for university entrance/completion of undergraduate studies has the worst LE in our analysis. This group would also probably show the most significant reduction in LE when affected by a disease [21]. In our dataset, the limited number of cases did not make an estimation of LE within the group of patients with diabetes or myocardial infarction possible. This remains an important topic for future research.

The value of the estimated mean life expectancy is higher than the mean LE reported by the 2002/2004 life table for Germany (men: 75.89; women: 81.55) and by the Bavarian life table, whose values (men: 76.47; women: 81.92) are the second highest among the German states. Our estimation remains slightly higher, even after correction of the left data truncation. However, this hardly affects the study results, whose major purpose is to show a gradient among different socio-economic groups and not to calculate a precise estimate of the LE of a newborn baby, if the current death rates continue to apply throughout his or her life. A possible reason for the difference resides in the small number of old and deceased people in the available dataset. This is particularly true in the case of women where the number of deceased participants (522) is almost half compared with men (1032), implying that the survival data of women is more heavily censored. Another reason could also lie in an over-representation of health conscious persons in the dataset, as health conscious persons are, presumably, more inclined to participate in a study dealing with health issues, and having a healthier lifestyle, they might live longer.

Most 95% confidence limits of the LE value overlap with other groups. This is largely due to the relatively small number of deceased people in the available data. However, it should be noted that the degree of overlap between the confidence intervals does not directly quantify the statistical significance regarding the *existence of a difference *between the life expectancies of the different groups.

The use of a parametric method was appropriate for the present analysis. Parametric methods are particularly useful to investigate small changes induced in the distribution function by certain data variables (e.g. sex, socio-economic status in the case of life expectancy). Also, they are more powerful than non-parametric methods for sparse data, provided the assumed shape for the underlying distribution function is a reasonable description of the data. The principal advantage of non-parametric methods is that they are free of any assumptions regarding the underlying distribution function, which protects against potential biases in the results if a parametric method is used with a distribution function that is actually a poor match for the data. However, the larger freedom regarding possible distribution functions explored by non-parametric methods means that datasets need to be very large to obtain accurate results with this method. Also, it is often difficult to extrapolate the estimated non-parametric distribution function into regimes that are not well sampled by the data. This is for example the case in building a life table with the MONICA/KORA data; the tail of the age distribution is in fact not well determined by non-parametric methods due to the small number of deceased people in the available data.

The small sample was clearly a limitation of this analysis and biased the estimates of LE. The inclusion in the survey of only people aged between 25-74 years and the absence of people living in institutions also limited a precise quantification of life expectancy. The survey is also limited by the regional data collection and possible recruitment bias.

While these limitations did not allow a robust estimation of absolute life expectancy, the results of the study confirmed, as have other German and international studies, the existence of a socio-economic gap in life expectancy. The study also provides important new information by addressing a public health topic rarely discussed to date, i.e. the socio-economic differences in the relative reduction of LE of people who have diabetes mellitus or myocardial infarction.

## Conclusions

This study confirms the existence of a socio-economic gap in LE in Germany and shows that the differences in LE by socio-economic status are comparable to those found in other European countries. It also measures, for the first time, the differences in the reduction of LE of persons who have diabetes mellitus or myocardial infarction for different income groups. In most cases the LE differences seem to increase when one of these diseases is present, indicating that the German health care system is not successful in reducing the LE differences among these patients. It would be reasonable to claim that the health care system should be able to reduce health inequalities among the patients, as patients with a higher need for health care (e.g. those in a lower income group) should receive more health care. The opposite seems to be the case, though, and it could be concluded that in the health care system more resources should be directed towards lower socio-economic status patients.

Concerning the statistical methods proposed here, the description of this method could help in making the estimation of the socio-economic gap in countries without a data-linkage system among the entries in public registers, such as Germany, more accessible.

## Competing interests

The authors declare that they have no competing interests.

## Authors' contributions

LP wrote the paper, performed the statistical analysis, and participated in the design of the study. UTS provided critical feedback on the statistical analysis and drafts. KHL and CM provided insights on the dataset and commented on drafts. AM designed the study, contributed to the writing and commented on drafts. All authors read and approved the final manuscript.

## Pre-publication history

The pre-publication history for this paper can be accessed here:

http://www.biomedcentral.com/1471-2458/10/135/prepub
